# Biomechanics of the tether breakage: tensile behaviour of a single-unit vertebral body tethering construct

**DOI:** 10.1007/s43390-023-00657-2

**Published:** 2023-02-10

**Authors:** Ogulcan Guldeniz, Christopher C. H. Yip, Wanis Nafo, Kenneth M. C. Cheung

**Affiliations:** 1 Department of Orthopaedic Surgery, HKU-Shenzhen Hospital, Shenzhen, China; 2 Department of Orthopaedics and Traumatology, The University of Hong Kong, Pokfulam, Hong Kong SAR, China; 3 Department of Mechanical System Engineering, Jeonbuk National University, Jeonju, Republic of Korea

**Keywords:** Vertebral body tethering, Adolescent idiopathic scoliosis, Tether breakage, Tensile testing

## Abstract

**Purpose:**

Tether breakage was reported as the most common complication of vertebral body tethering. However, as the literature suggests the physiological loads do not have the potential to cause the failure of the tether. Currently, the biomechanical reason behind the tether breakage is unknown. The current study aims to elucidate the effects of the tension forces on the failure mechanisms of the VBT and provide mechanical justification for how it can be identified radiographically.

**Methods:**

Tensile tests (20%/min strain rate) were performed on single-unit VBT samples. Failure modes and mechanical characteristics were reported.

**Results:**

The failure took place prematurely due to the slippage of the tether at the screw–tether junction where the tether is damaged significantly by the locking cap. Slippage was initiated at 10–13% tensile strain level where the tensile stress and tension force were 50.4 ± 1.5 MPa and 582.2 ± 30.8 N, respectively.

**Conclusion:**

The failure occurs because of high-stress concentrations generated within the locking region which damages the tether surface and leads to the slippage of the tether. We observed that the loads leading to failure are within the physiological limits and may indicate the high likelihood of the tether breakage. The failure mode observed in our study is shown to be the dominant failure mode, and a design improvement on the gripping mechanism is suggested to avoid failure at the screw–tether junction. We observed that the tether elongates 10–13% prior to the breakage, which can be employed as a diagnostic criterion to screen for tether breakages radiographically.

## Introduction

Adolescent idiopathic scoliosis (AIS) is a subset of paediatric patients with scoliosis, defined by the diagnosis of the deformity in children over the age of 10 years until skeletal maturity [1]. AIS is reported to be associated with increased back pain [7], a decreased range of motion [8] and poor cosmesis, and can lead to impaired pulmonary function, which progresses into adulthood [9, 10]. Currently, AIS is one of the most common spinal deformities in the world that paediatric orthopaedic surgeons treat regularly [11]. There is no curative treatment for AIS, as the aetiology of the deformity is unknown [12].

Traditionally spinal fusion has been the only surgical option to achieve correction of the curve [2]. However, spinal fusion completed before the spinal growth would come at the price of restricted spinal mobility, arrests growth on the fused sites, and leads to poor respiratory development [3–6]. To avoid the growth-related complications of spinal fusion and allow the natural growth of the spine to accomplish the correction growth modulation, spinal instruments such as Magnetically Controlled Growing Rods were introduced [7]. However, the treatment procedure employing such instruments still comes with an aimed final fusion [8].

Vertebral body tethering, a novel growth modulation method, has gained increasing popularity over the past decade. The procedure aims to achieve progressive curve correction in AIS patients whilst avoiding fusion [9]. However, according to several clinical studies, complications of VBT are not uncommon; in their recent meta-analysis, Shin et al. reported a complication rate of 26% and a revision rate of 24.7%. Moreover, they reported that the capabilities of VBT deteriorate, leading to an increased complication rate over time. Tether breakage was reported as the most common complication associated with VBT [10]. Trobisch et al. reported the breakage rate as 24% for single tether segments and 16% for double tether segments for the lumbar curve [11]. The same was reported as 52% for the thoracic curve by Newton et al. [12], and 48% by Hoernschemeyer et al. [13]. On the other hand, Alanay et al. reported the breakage rate as only 3% [14]. The reason behind such variation was speculated to be due to different surgical techniques [11].

The base material of tether, PET, has been reported to be stronger than some conventional types of steel, and its highly elastic nature is well known [15, 16]. Failure stress of the PET under tensile loading was reported as 736–1260 MPa in the literature, which demonstrated a high variation between the results due to manufacturing- and testing-related factors such as strain rate, monomer direction, etc. [15, 17, 18]. However, recent biomechanical studies investigating the loads on the spine show that the highest physiological loads reach only up to approximately 650 N on the L4/L5 level, which corresponds to only 52 MPa (assuming the loads are coinciding with the tether’s axis) [19]. Furthermore, recent studies have shown that a correction force of 150–200 N is sufficient to modify the asymmetrical compression in AIS patients [20]. Thus, the occurrence of physiological loads high enough to cause the tensile failure of the PET is highly unlikely. Currently, to the authors’ knowledge, the biomechanical reason behind why the tether breakage occurs so easily is unknown.

The objective of this work was to elucidate the structural mechanics and the failure mechanisms of the VBT tether to better understand the causes of tether breakage and to provide mechanical justification for how tether failure on radiographs can be defined. For that purpose, we performed tensile tests on single-unit VBT constructs and reported our findings. We interpreted our data considering the loads applied during the surgical procedure and the body’s natural load-applying capacity. We believe our findings will be crucial in identifying the tether breakages in the future, contribute immensely to the research and development of the VBT, and will influence future modifications in the surgical technique of the VBT.

## Materials and methods

### Sample preparation

**Fig. 1:**
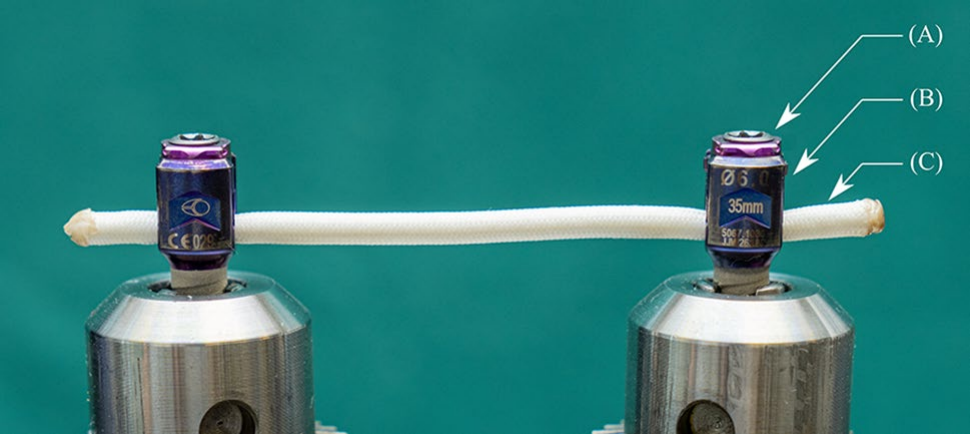
Test sample, where the locking caps (**A**), the screws (**B**), and the tether (**C**) are shown

Single-unit VBT samples were prepared using REFLECT^™^, Scoliosis Correction System (Globus Medical, USA). Each tether test sample was placed between a pair of screws and locked into position according to the surgical technique described by the manufacturer (Fig. 1).

The diameter of the tether samples was measured as 4 mm, whilst the gauge length was standardised to 60 mm for the purpose of testing considering the reported approximate size of a single functional unit [21–23].

### Tensile test procedure

**Fig. 2:**
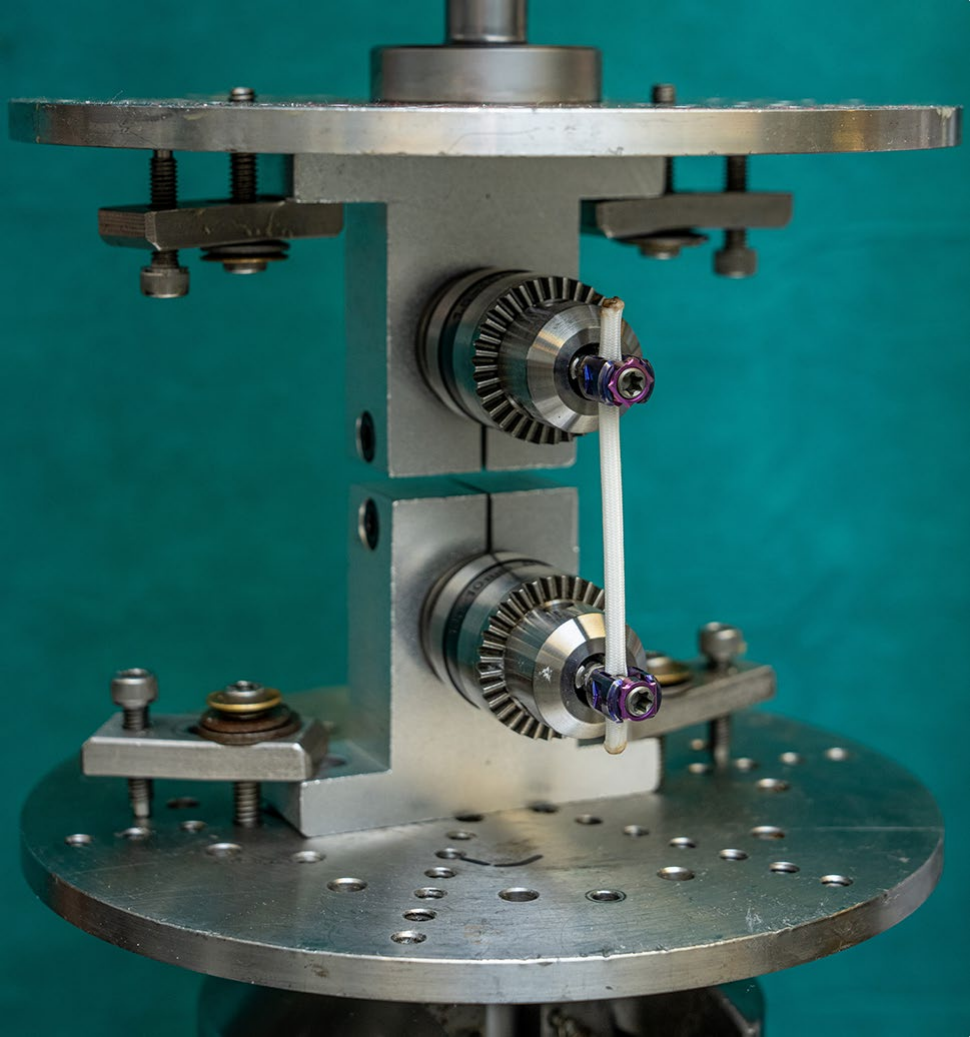
The custom-made clamping system

A mechanical testing frame (MTS, 858 Mini Bionix) was used to conduct the tensile tests. The device consists of two mechanical clamps, a hydraulic motor system, and a 1 kN load cell (20 Hz sampling rate). The loading capacity of the hydraulic motor system ranges from 100 to 30,000 N with a sampling rate of up to 1000 Hz. To mimic the in vivo tensile loading biomechanics, a custom-made clamping system was designed and mounted to the MTS machine to grip the screws perpendicular to the axis of the tensile loading direction and to make sure there is no rotational difference between the screw heads during the testing which may lead to stress concentrations between the screw and the tether [24] (Fig. 2).

Five samples were tested. The samples were loaded at a strain rate of 20% per minute [15, 16, 25, 26]. Tests were terminated when the 25% strain threshold was reached. Similar to the literature, as the stiffness of the base material of the tether, PET, is significantly lower compared to that of the loading system, the displacement recorded by the MTS was accepted as the deformation of the samples [18, 25]. All tests were performed at room temperature (approximately 20 °C), and approximately 50% relative humidity. No significant loss in strength of PET after clinical implantation was reported in the literature [27]. Load and displacement data were collected during the tests and analysed.

## Results

### Failure mode

**Fig. 3:**
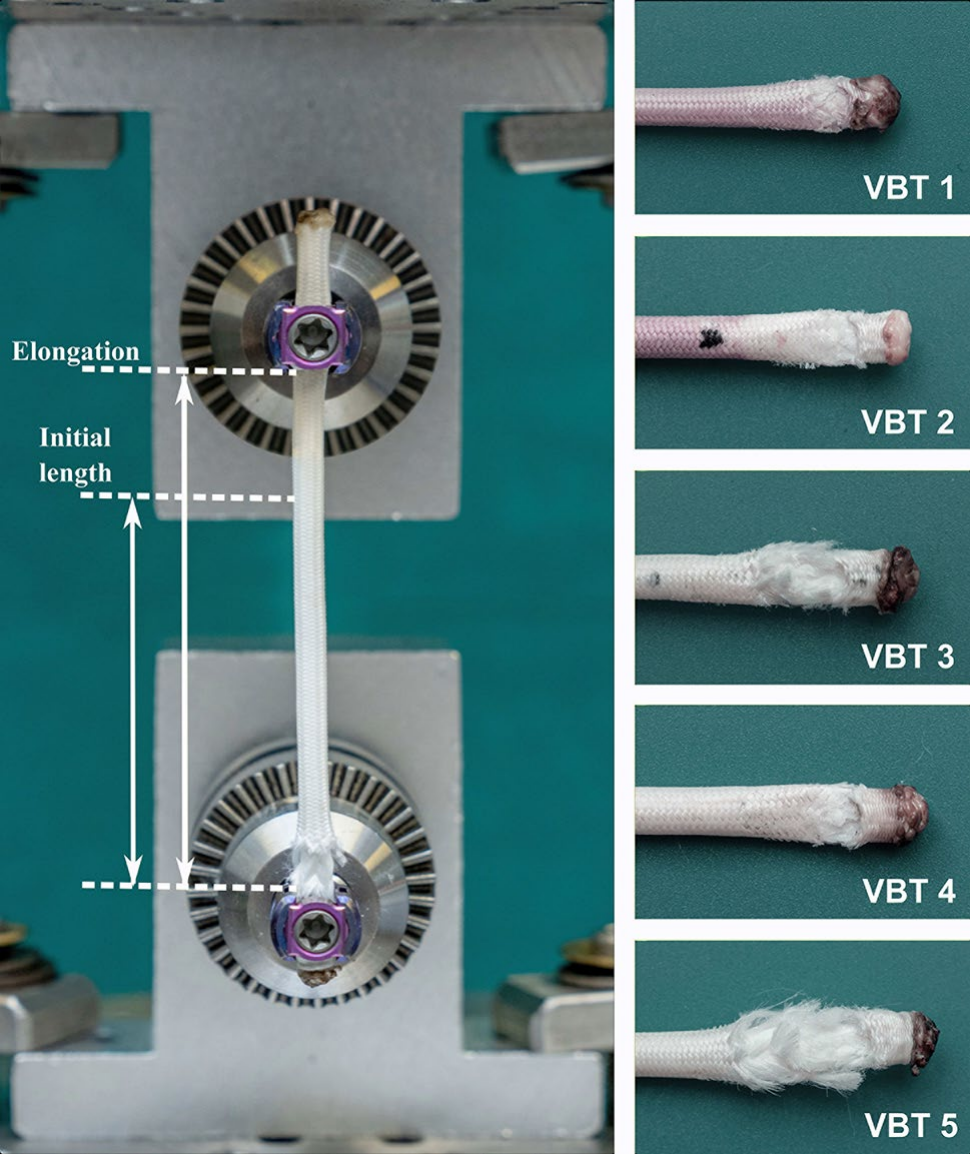
An image of a sample right after the experiment where initial length and the elongated length are illustrated (left), and the failure modes of all the tether samples at the screw–tether junction (right)

Tensile failure was not observed in our tests as the failure occurred at the screw–tether junction due to complex loads generated by the gripping mechanism of the screw, which was followed by the slippage of the tether. The reason for slippage was observed to be the decrease in the cross-sectional area of the tether caused by the local damage at the screw–tether junction. Failure modes of the tether samples under tensile loading are shown in Fig. 3. The same failure mechanism was observed in all five samples. The failure occurred at the cranial screw–tether junction for three of the samples, whereas two of them failed at the caudal screw–tether junction.

### Experimental results

**Fig. 4:**
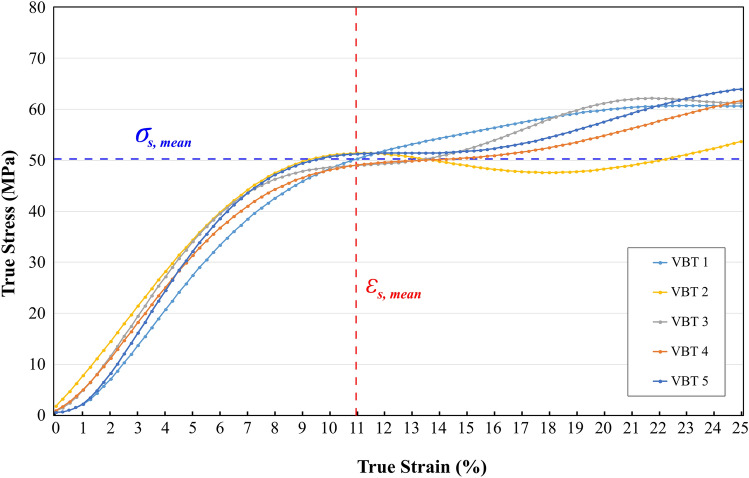
True stress–true strain curves from the tensile tests

**Fig. 5:**
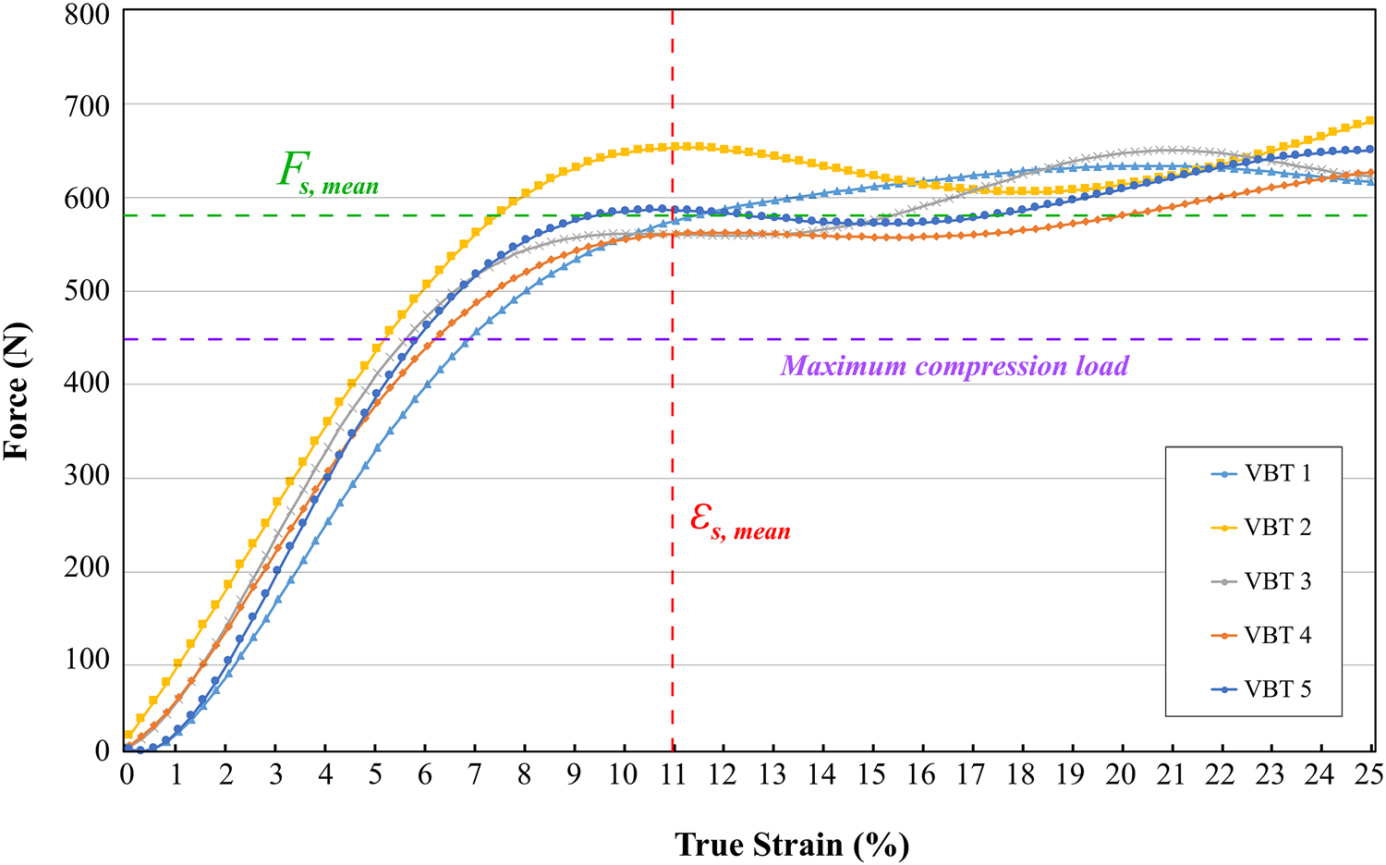
Force–true strain curves from the tensile tests

True stress–true strain curves and force–true strain curves are shown in Figs. 4 and 5, respectively. Force–true strain curves were reported for further comparison as the surgical technique involves force input to generate segmental compression. As all the samples were sharing the same geometrical characteristics, a direct comparison between the force responses was possible without normalising the data.

The mechanical characteristics of the samples are given in Table 1. As the failure occurs at the screw–tether junction with the slippage of the tether, the values represent the onset of slippage at the screw–tether junction. Slippage on the screw–tether junction was initiated when a 10–13% strain threshold was reached. On average, the slippage stress and strain values were measured as 50.4 ± 1.5 MPa and 11.2 ± 0.9%, respectively.

**Table 1:** Slippage stress, strain, and force values of all the tether samples and their means and standard deviations

	VBT 1	VBT 2	VBT 3	VBT 4	VBT 5	Mean
Slippage stress, ${\sigma }_{s}$ (MPa)	52.2	51.3	48.4	48.8	51.1	50.4 ± 1.5
Slippage strain, ${\varepsilon }_{s}$ (%)	13	11	10	11	11	11.2 ± 0.9
Slippage force, ${F}_{s}$ (N)	582.5	644.9	552.8	552.4	578.6	582.2 ± 30.8

The mean applied force when slippage occurred was only 582.2 ± 30.8 N. Surprisingly, if the calibration on the manufacturer’s compression device accurately represents the amount of force applied to the tether during compression, it was only 132.2 N higher than the maximum 450 N initial load applied to the tether during surgery. The overall behaviour of the samples was similar and revealed a non-linear behaviour.

## Discussion

VBT has emerged over the past decade as an alternative non-fusion technique for treating AIS patients [28]. Compared to spinal fusion, VBT accomplished the preservation of the spinal motion, whilst allowing the natural growth of the spine to correct the curve gradually [8]. Nevertheless, despite all the potential benefits, tether breakage was reported as the most common complication of VBT after surgery with an occurrence rate of up to 52% [11–14]. To the authors’ knowledge, the biomechanical reason behind why the tether breakage occurs so easily is unknown [10]. In this study, to understand the structural mechanics and the failure mechanisms of the VBT, we performed tensile tests on single-unit VBT constructs (REFLECT^™^, Scoliosis Correction System, Globus Medical), and reported our findings. We interpreted our data considering the loads applied during the surgical procedure and the body’s natural load-applying capacity.

To the authors’ knowledge, this is the first study investigating the tensile response of the VBT, and the first attempting to address the underlying mechanisms of the tether breakage in an interdisciplinary manner employing both clinical and mechanical approaches.

Failure at the gripping region is a well-known problem of mechanical testing as the stress concentrations generated by the complex loads prevent accomplishing pure tensile loads; therefore, prevent accomplishing tensile failure, which eventually leads to premature failure under tensile loads that are significantly lower than the ultimate tensile stress of the material [29, 30]. Similarly, tensile failure was not observed in our tests as the failure occurred at the screw–tether junction due to complex loads generated by the gripping mechanism of the screw, which was followed by the slippage of the tether. The average slippage stress reported in our study (50.4 ± 1.5 MPa) only corresponded to 4–7% of the ultimate tensile stress of the PET (736–1260 MPa) [15, 17, 18]. Premature failure at the screw–tether junction and a decrease in the lifetime of the tether due to complex loading at the screw–tether junction has been reported before by Nafo et al. for the malaligned levels, and our results are in line with their findings [24]. Our results suggest that a design improvement on the gripping mechanism of the REFLECT^™^ screws is necessary to avoid the generation of complex loads at the screw–tether junction, which is expected to improve the overall gripping performance and prevent premature failure of the tether.

Our results show that the failure mode observed in our study which occurred due to the complex loads generated at the screw–tether junction is the dominant mode of failure, as the failure occurs prematurely before high enough tensile loads are accomplished for the other failure modes to take place. Lechat et al. reported the behaviour of PET under cyclic loading to investigate the fatigue behaviour of the PET and showed that the lifetime of PET is 1e8 cycles for a cyclic load fluctuating between 0 and 55% of the ultimate tensile stress [17]. Similarly, they also reported the creep behaviour of PET and observed creep failure only at 80% of the ultimate tensile load [15]. Compared to the reported data in the literature, our results indicate that the occurrence of other modes of failure is highly unlikely as these failure modes require at least 55% of the ultimate tensile load to be accomplished, whereas our results show that the REFLECT^™^ VBT system can only reach up to 4–7% of the ultimate tensile load of PET.

The most common method used for radiologic diagnosis of tether breakage is an increase in the inter-screw angle of 5 degrees [11–14]. However, this would assume that breakages result in a loss of correction, which is shown to be not always the case, and explain why a change in inter-screw angle is recognized to underdiagnose tether breakages [31, 32]. We observed that the tether elongates 10–13% before the failure occurs, where the strain values represent this elongation between any two adjacent screw heads in percentage. Contrary to other mechanical parameters, the strain has the potential to be the bridge between any mechanical investigation and radiographical examination as the measurement of elongation between the post-op and follow-up radiographs is possible. Thus, we propose the use of an increase in inter-screw distance of more than 13% of its original length to diagnose the tether breakages radiographically as an alternative to the inter-screw angle. However, further clinical studies are necessary to establish this as a reliable diagnosis method of tether breakage. Compared to the conventional inter-screw angle, inter-screw distance does not assume that the breakages result in a loss of correction.

The slippage force values reported in our work were significantly close to the reported physiological loads in the literature, which could explain the tether breakage in vivo. In a recent study, intervertebral disc reaction forces during a 30-degree lateral bending were reported as 453 N, 460 N, and 652 N on the T6/T7, T12/L1, and L4/L5, respectively [19]. In another study, disc compressive forces during a 30-degree lateral bending on T11/T12 and L4/L5 were reported as 100% and 130% of the body weight, respectively, which corresponds to approximately 320–750 N for a 10–20-year-old adolescent (average 32–58 kg weight) [33]. Similarly, in another study, forces applied by the external oblique muscle during lateral bending were reported as approximately 300 N for adult male volunteers [34]. Although these loads do not represent the actual tensile loads applied to the tether, due to the nature of lateral bending, compressive forces can be interpreted as tensile loads on the stretched side of the body and can be used as a reference of the physiological load-applying potential of the spine and the surrounding tissues.

The maximum compression force of 450 N that was applied to the tether during the surgery by the compressor instruments of REFLECT^™^ was only 132.2 N lower than the average slippage force we reported in our study, which may indicate that the introduction of high compression forces for the initial correction of the curvature may increase the likelihood of failure at those levels. However, this would only hold true if the loads are stored within the material as internal stresses. Considering the viscoelastic nature of the PET generation of internal stresses is unlikely as the compressive loads are expected to relax in time [35]. Further study is suggested to investigate the relaxation behaviour of the PET tether under varying compression forces.

It should be noted that our results are a product of a controlled testing setup which is constrained systematically to prevent the involvement of other clinical factors such as malalignment and rotational differences between the adjacent screws, as these factors may increase the contact interaction between the screw and the tether, and increase the likelihood of occurrence of high-stress concentrations [24]. Thus, the slippage loads reported in our study are expected to be even lower in instrumented VBT constructs due to such factors, and further study is necessary to further elaborate the influence of such clinical factors.

A limitation of the current study is that our results only represent the behaviour of only one VBT system. As the mechanical properties of the PET have shown to be varying significantly due to various reasons and the effects of the gripping mechanism are well established in our study [15, 17, 18], the mechanical behaviour of the other VBT systems is expected to be different. Therefore, these systems also need to be studied in a similar manner to understand their failure mechanisms and other potential mechanical complications. Such studies would also be beneficial in identifying the inter-screw distance cut-off values for those VBT systems for the diagnosis of tether breakage radiographically.

In summary, our results showed that the failure occurs as a result of high-stress concentrations generated at the screw–tether junction, which damages the outer surface of the tether in that area and leads to the slippage of the tether. We observed that the loads leading to failure are within the bodily load limits, which may indicate the high likelihood of tether breakage. Our results suggest that the failure mode observed in our study is the dominant failure mode, and a design improvement on the gripping mechanism of the REFLECT^™^ screws is necessary to avoid the generation of complex loads at the screw–tether junction. We propose the use of inter-screw distance as a more mechanically accurate criterion to identify the tether breakages radiographically. We believe our findings will be crucial in identifying the tether breakages in the future, contribute immensely to the research and development of the VBT, and will influence future modifications of the surgical technique of the VBT.

## Data Availability

The datasets generated and/or analyzed during the current study are available from the corresponding author upon request.
